# Hydrogen sulfide attenuates cytokine production through the modulation of chromatin remodeling

**DOI:** 10.3892/ijmm.2015.2176

**Published:** 2015-04-08

**Authors:** ESTER C.S. RIOS, BARTOSZ SZCZESNY, FRANCISCO G. SORIANO, GABOR OLAH, CSABA SZABO

**Affiliations:** 1 Department of Anesthesiology, University of Texas Medical Branch, Galveston, TX, USA; 2 Department of Emergency Medicine, University of São Paulo Medical School, São Paulo, Brazil

**Keywords:** histone deacetylase, inflammation, hydrogen sulfide, endotoxin

## Abstract

Hydrogen sulfide (H_2_S) is an endogenous gaseous biological mediator, which regulates, among others, the oxidative balance of cells under normal physiological conditions, as well as in various diseases. Several previous studies have reported that H_2_S attenuates inflammatory mediator production. In this study, we investigated the role of H_2_S in chromatin modulation in an *in vitro* model of lipopolysaccharide (LPS)-induced inflammation and evaluated its effects on inflammatory cytokine production. Tamm-Horsfall protein 1 (THP-1) differentiated macrophages were pre-treated with sodium hydrosulfide (NaHS) (an H_2_S donor) at 0.01, 0.1, 0.5 or 1 mM for 30 min. To stimulate cytokine production, the cells were challenged with bacterial LPS (1 *μ*g/ml) for 1, 4, 8 or 24 h. Histone H3 acetylation was analyzed by chromatin immunoprecipitation (ChIP), cytokine production was measured by ELISA and histone deacetylase (HDAC) activity was analyzed using a standard biochemical assay. H_2_S inhibited the production of interleukin-6 (IL-6) and tumor necrosis factor-α (TNF-α) in a concentration-dependent manner; it was most effective at the two highest concentrations used. This effect was associated with a decrease in histone H3 acetylation at the IL-6 and TNF-α promoters in the cells exposed to H_2_S or H_2_S + LPS. The findings of the present study suggest that H_2_S suppresses histone acetylation, which, in turn, inhibits chromatin openness, leading to a decrease in the gene transcription of various pro-inflammatory cytokines. Therefore, this mechanism may contribute to the previously demonstrated anti-inflammatory effects of H_2_S and various H_2_S donors.

## Introduction

Hydrogen sulfide (H_2_S) is an endogenous gaseous mediator with regulatory roles in neurotransmission, cardiovascular function and cell metabolism. It also participates in the regulation of the oxidative balance of the cells, under both normal physiological conditions, as well as in various diseases (1–8). Various classes of H_2_S donors have been tested in multiple models of inflammation. The results have revealed that H_2_S exerts cytoprotective and anti-inflammatory effects, including the inhibition of multiple pro-inflammatory signaling pathways and a reduction in the production of reactive oxygen and nitrogen species (9–24).

The post-translational modification of histones is one form of epigenetic modifications that alter gene expression (25). Amino acids present in the histone tail can be modified by acetylation, methylation, phosphorylation, ubiquitination and other enzymatic modifications during RNA synthesis (26). Histone acetylation is associated with chromatin unfolding, i.e., it facilitates gene transcription. On the other hand, histone deacetylation inhibits gene transcription. Histone methylation can either inhibit or activate gene transcription, depending on the localization (27). Histone methyltransferases (HMTs, enzymes that transfer acetyl groups to the histone tail at lysine and arginine residues) promote histone methylation, while histone acetylation is mediated by histone acetyltransferases (HATs) that exert their effects at lysines of histones H3 and H4 (26–28). Neither histone methylation nor acetylation is permanent, as the modifications can be removed by histone deacetylases (HDACs) and demethylases, respectively, thus rendering epigenetic regulation a dynamic regulator of gene transcription (29). In the present study, we investigated whether H_2_S acts as a regulator of chromatin modulation and cytokine production in an *in vitro* model of inflammation.

## Materials and methods

### Cell culture

Tamm-Horsfall protein 1 (THP-1) cells were maintained in RPMI-1640 supplemented with 2 mm l-glutamine, 100 U/ml penicillin, 100 *μ*g/ml streptomycin and 10% fetal bovine serum (FBS; Sigma, St. Louis, MO, USA). Ultrapure *Escherichia coli* 0111:B4 LPS free of lipoproteins was obtained from Invitrogen (San Diego, CA, USA). The cells were plated in 22-mm tissue culture dishes (2×10^6^ cells/dish). Macrophage differentiation was induced with phorbol myristate acetate (PMA, 100 nM) for 5 h. In one set of experiments (pre-treatment experiments) the effects of H_2_S were examined following a 30-min pre-treatment with sodium hydrosulfide (NaHS, an H_2_S donor) (Sigma) at 0.01, 0.1, 0.5 or 1 mM followed by a washout, followed by incubation with bacterial lipopolysaccharide (LPS, 1 *μ*g/ml) in 1% FSB RPMI-1640 for 1, 4, 8 or 24 h. In another set of experiments (co-treatment experiments), NaHS was administered 30 min prior to the LPS administration, without a washout. The control cells were maintained in 1% FBS RPMI-1640.

### Western blot analysis

The cells were placed in RIPA buffer and sonicated (3 times for 10 sec each). The supernatants were preserved and the protein concentration was determined by bicinchoninic acid (BCA) assay. Protein expression was determined by sodium dodecyl sulfate-polyacrylamide gel electrophoresis (SDS-PAGE) under reducing conditions. Cell extracts (25 *μ*g/ml) were boiled in equal volumes of loading buffer (150 mM Tris-HCl, pH 6.8; 4% SDS; 20% glycerol; 15% β-mercaptoethanol; and 0.01% bromophenol blue) and were electrophoresed on 8–12% polyacrylamide gels. Following electrophoretic separation, the proteins were transferred onto PVDF membranes for western blotting. The membranes were blocked with StartingBlock T20 (PBS) Blocking Buffer (Thermo Scientific, Waltham, MA, USA) for 1 h. The following primary antibodies were used: rabbit acetylated histone H3 at the N-terminal tail (06-599; Millipore, Billerica, MA, USA), trimethyl-histone H3 at lysine (Lys)9 (17-625; Millipore), trimethyl-histone H3 at Lys27 (17-625; Millipore), and HRP-conjugated β-actin (Santa Cruz Biotechnology, Inc., Santa Cruz, CA, USA). The primary antibodies were incubated overnight at 4°C and the membranes were washed twice in TBST. A secondary horseradish peroxidase-conjugated antibody (goat anti-rabbit; Cell Signaling Technology, Danvers, MA, USA) was then applied at a dilution of 1:5,000 for 1 h. Over a 30-min period, the blots were washed twice in TBST, after which they were incubated in enhanced chemiluminescence reagents (SuperSignal Detection kit; Pierce, Rockford, IL, USA). The band intensity of the original blots was quantified using GeneTools (Syngene; Synoptics Ltd., Cambridge, MA, USA) and was normalized to β-actin expression.

### Chromatin immunoprecipitation (ChIP)

Chromatin immunoprecipitation was performed using the EZ-ChIP kit following the manufacturer’s instructions (17-371; Millipore). Following stimulation, the THP-1 cells were fixed by the addition of 37% formaldehyde to a final concentration of 1%. After 10 min, 10X glycine was added. The cells were washed with ice-cold phosphate-buffered saline (PBS), collected and centrifuged for 4 min at 700 × g. The cells were then lysed with SDS lysis buffer. Chromatin was sheared by sonication (5×10 sec at approximately 30% of maximum power), centrifuged to pellet debris and in dilution buffer. Chromatin extracts were pre- cleared for 1 h with a 50% suspension of protein G agarose. Immunoprecipitations were carried out overnight at 4°C with the following antibodies: ChIPAb trimethyl-histone H3 at Lys9 (17-625; Millipore), ChIPAb trimethyl-histone H3 at Lys27 (17-625; Millipore) and acetylated histone H3 at the N-terminal tail (06-599; Millipore). Immune complexes were collected with protein G for 1 h and washed 3 times with high-salt buffer (20 mM Tris at pH 8.0, 0.1% SDS, 1% NP-40, 2 mM EDTA and 0.5 M NaCl) followed by washes in low-salt buffer (50 mM NaCl) and no salt buffer (TE). Immune complexes were extracted in elution buffer and DNA cross-links were reverted by heating at 65°C for 12 h. Following proteinase K digestion, DNA was extracted using spin columns following the manufacturer’s instructions. The following promoter-specific primers were used in the polymerase chain reactions (PCRs): TNF-α forward, 5′-GATTCTGAGCAAAATA GCCAGCA-3′ and reverse, 5′-GGCTTCCTTCTTGTTG TGTGT-3′; interleukin-6 (IL-6) forward, 5′-CCTAGTTGT GTCTTGCGATG-3′ and reverse, 5′-GGAGGGGAGATAG AGCTTCT-3′.

### Measurement of cytokine production and HDAC/HAT activity

The medium was collected to determine the levels of tumor necrosis factor-α (TNF-α) and IL-6 using ELISA kits (R&D Systems, Minneapolis, MN, USA). HDAC activity was analyzed in the cell extracts by a colorimetric assay (HDAC activity assay kit K331-100; BioVision, Mountain View, CA, USA). As negative control, we added Trichostatin (TSA) to the THP-1 extract at final concentration of 0.01 mM following the manufacturer’s instructions. HAT activity was also analyzed in the cell extract using a histone acetyltransferase activity assay (ab65352). All procedures were conducted according to the manufacturer’s recommendations.

### Statistical analysis

All values are expressed as the means ± standard error of the mean (SEM) from 5 or 6 repetitions per group for the biochemistry analysis and 3–4 technical replicates for the western blot analyses. Statistical analysis was performed using GraphPad InStat software (GraphPad Software Inc., San Diego, CA, USA). Comparisons among the experimental groups were carried out by analysis of variance and Tukey’s post-hoc test. A p-value <0.05 was considered to indicate a statistically significant difference.

## Results

### H_2_S attenuates cytokine production and modulates HDAC activity

**Figure 1:**
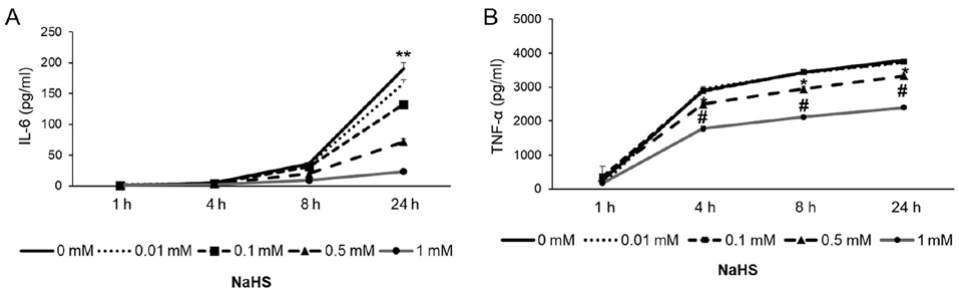
Effect of pre-treatment with hydrogen sulfide (H_2_S) on lipopolysaccharide (LPS)-induced cytokine production. Differentiated THP-1 macrophages were pre-treated with various concentrations of sodium hydrosulfide (NaHS), for 30 min, followed by a washout, and subsequently challenged with LPS (1 *μ*g/ml). The levels of (A) interleukin-6 (IL-6) and (B) tumor necrosis factor-α (TNF-α) in the supernatant were analyzed at 1, 4, 8 and 24 h post-LPS challenge. NaHS exerted an inhibitory effect on cytokine production. ^*^p<0.05 vs. 0, 0.01 and 0.1 mM; ^**^p<0.05 vs. all groups; ^#^p<0.05 vs. all groups.

**Figure 2:**
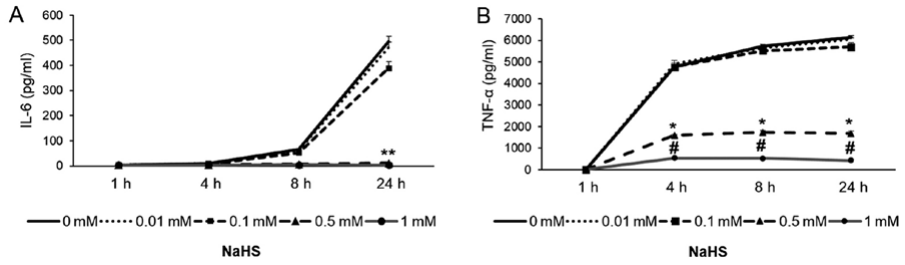
Effect of treatment with hydrogen sulfide (H_2_S) on lipopolysaccharide (LPS)-induced cytokine production. Differentiated THP-1 macrophages were treated with various concentrations of sodium hydrosulfide (NaHS) for 30 min, followed by LPS challenge (1 *μ*g/ml). The levels of (A) interleukin-6 (IL-6) and (B) tumor necrosis factor-α (TNF-α) in the supernatant were analyzed at 1, 4, 8 and 24 h post-LPS challenge. NaHS exerted an inhibitory effect on cytokine production. ^*^p<0.05 vs. 0, 0.01 and 0.1 mM; ^**^p<0.05 vs. all groups; ^#^p<0.05 vs. all groups.

**Figure 3:**
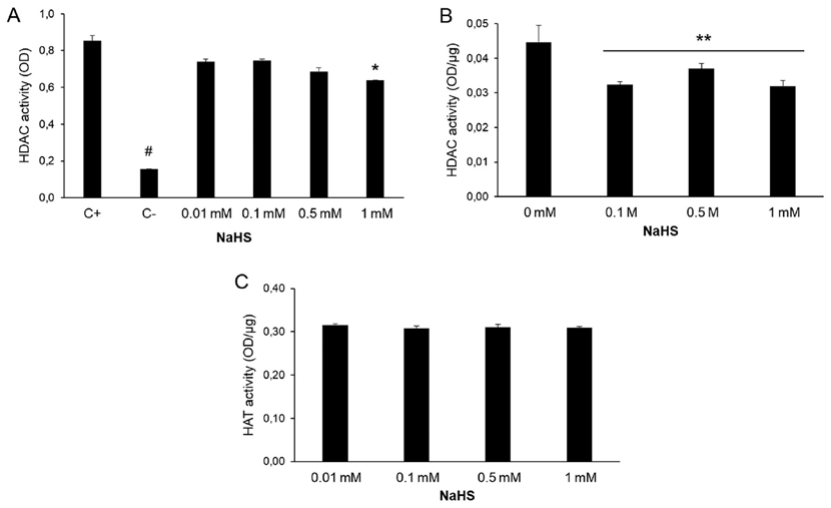
Effect of hydrogen sulfide (H_2_S) on histone deacetylase (HDAC) and histone acetyltransferase (HAT) activity. (A) HDAC activity in THP-1 extracts treated with various concentrations of sodium hydrosulfide (NaHS) for 30 min. Trichostatin (TSA; C^−^) was used as a positive control. (B) HDAC activity was measured in the THP-1 cells pre-treated with NaHS for 30 min, followed by a challenge with lipopolysaccharide (LPS) (1 *μ*g/ml) for 4 h. Note the lack of effect of NaHS alone (A), in contrast to the effect of NaHS pre-treatment (B), when applied prior to LPS challenge. (C) HAT activity was measured in THP-1 cells pretreated with NaHS for 30 min, followed by a challenge with LPS (1 *μ*g/ml) for 4 h. ^*^p<0.05 vs. C^+^, 0.01 and 0.1 mM; ^#^p<0.05 vs. all groups; ^**^p<0.05 vs. 0 mM.

**Figure 4:**
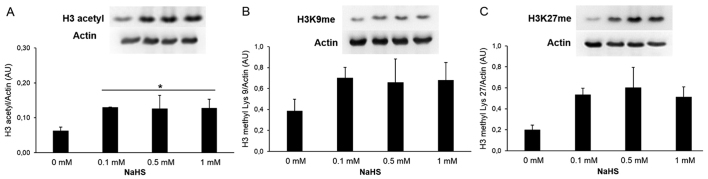
Modification of histone H3 by hydrogen sulfide (H_2_S). The effect of sodium hydrosulfide (NaHS) on histone H3 (A) acetylation, (B) methylation at lysine (Lys)9 and (C) methylation at Lys27 was analyzed in differentiated THP-1 cells followed by treatment with NaHS for 30 min. Cells were harvested at 4 h after the initiation of NaHS treatment for western blot analysis. Note the positive effect of NaHS on histone H3 modification. ^*^p<0.05 vs. 0 *μ*M.

**Figure 5:**
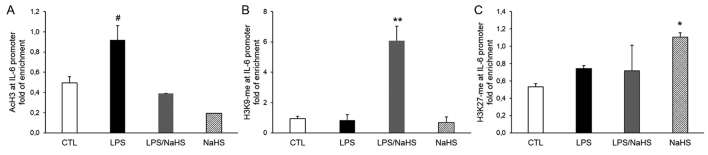
Hydrogen sulfide (H_2_S) affects lipopolysaccharide (LPS)-induced histone H3 modification at the promoter of interleukin-6 (IL-6). The (A) acetylation of histone H3 (AcH3), (B) histone H3 methylated at lysine (Lys)9 and (C) histone H3 methylated at Lys27 was analyzed by chromatin immuno precipitation (ChIP). Note that LPS alone increased the acetylation at the IL-6 gene promoter, which was attenuated by pre-treatment with sodium hydrosulfide (NaHS). In addition, pre-treatment with NaHS increased the methylation of histone H3 at the promoter of IL-6 at Lys9, but not at Lys27. ^*^p<0.05 vs. LPS; ^**^p<0.05 vs. control (CTL) and LPS; ^#^p<0.05 vs. all groups.

**Figure 6:**
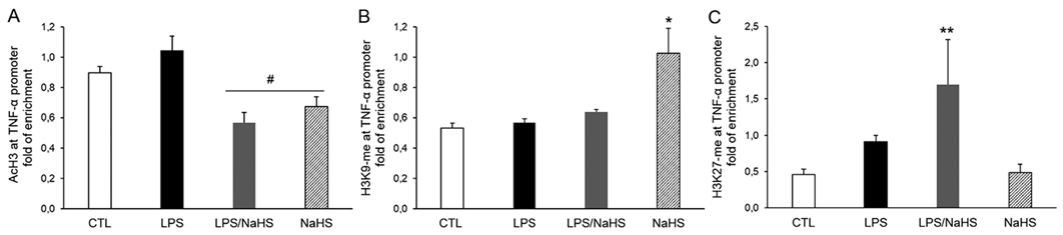
Hydrogen sulfide (H_2_S) affects lipopolysaccharide (LPS)-induced histone H3 modification at the promoter of tumor necrosis factor-α (TNF-α). The (A) acetylation of histone H3 (AcH3), (B) histone H3 methylated at lysine (Lys)9 and (C) histone H3 methylated at Lys27 was analyzed by chromatin immuno precipitation (ChIP). Pre-treatment with sodium hydrosulfide (NaHS) on its own or together with LPS challenge reduced the acetylation bound to the TNF-α gene promoter. NaHS increased the chromatin methylation at Lys9 but not at Lys27. LPS, on the one hand, increased the association of TNF-α with the methylation of histone H3 at Lys27 but not at Lys9. NaHS pre-treatment further increased the LPS-induced of methylation histone H3 at Lys27. ^*^p<0.05 vs. LPS; ^**^p<0.05 vs. all groups; ^#^p<0.05 vs. control (CTL) and NaHS.

Pre-treatment with NaHS inhibited the LPS-induced production of IL-6 and TNF-α in a concentration-dependent manner, as measured by ELISA (Figs. 1 and 2). The effects of NaHS on HDAC activity were analyzed in the THP-1 extracts (Fig. 3A) and in the macrophage cultures (Fig. 3B). HAT activity was analyzed in the macrophage cultures (Fig. 3C). NaHS reduced the activity of HDAC in the cell extracts (Fig. 3A). Moreover, the macrophages pre-treated with NaHS for 30 min exhibited a significant decrease in HDAC activity, as measured at 4 h (Fig. 3B). In contrast to HDAC activity, HAT activity was not affected by treatment with NaHS (Fig. 3C). H_2_S modulates histone acetylation and methylation, and regulates histone modifications at the IL-6 and TNF-α promoters. The effects of NaHS on chromatin were analyzed in the cells pre-treated with NaHS (0.1, 0.5 and 1 mM for 30 min, followed by stimulation with LPS (1 *μ*g/ml) for 4 h. Pre-treatment with the H_2_S donor increased the acetylation of histone H3 (Fig. 4A) and the methylation at Lys9 (Fig. 4B). The methylation of histone H3 at Lys27 did not present a statically significant change (Fig. 4C). The cells were also analyzed by the chromatin immunoprecipitation method. The chromatin associated with histone H3 (acetylated, methylated at Lys9 or Lys27) was precipitated prior to the determination of TNF-α and IL-6 gene expression. The values shown represent the enrichment of histone H3 acetylation or methylation compared to the input. The IL-6 (Fig. 5A) and TNF-α (Fig. 6A) promoters were associated with a lower histone H3 acetylation in the H_2_S-treated groups. H_2_S enriched the IL-6 (Fig. 5C) and TNF-α (Fig. 6B) promoters for histone H3 methylated at Lys27 and Lys9, respectively. On the other hand, the cells that were treated with both NaHS and LPS exhibited an enrichment in histone H3 methylation at Lys9 in the IL-6 promoter (Fig. 5B) and at Lys27 in the TNF-α promoter (Fig. 6C).

## Discussion

The results of the present study demonstrate that H_2_S modulates the acetylation and methylation of histones. Based on the known role of these epigenetic alterations in the regulation of pro-inflammatory mediator production (25–29), we hypothesized that these effects may contribute to a reduction in the amount of cytokines released following stimulation with LPS. Moreover, the present findings are also consistent with the conclusion that H_2_S, on its own, induces significant epigenetic alterations.

Given the short half-life of Na_2_S or NaHS in aqueous solutions (30), it is interesting to note that even a temporary stimulus (pre-treatment, followed by a washout) with the H_2_S donor NaHS reduces cytokine production and induces histone modifications. Our results revealed that the H_2_S donor increased the total methylation and acetylation of histone H3. Since HAT activity was not altered under the same conditions, it can be concluded that the effect of H_2_S on histone acetylation is due to the direct action of H_2_S on the reduction of HDAC activity, as demonstrated in cell culture and by a direct biochemical assay. It can also be concluded that, in turn, H_2_S reduces the chromatin openness by decreasing histone acetylation at the IL-6 and TNF-α promoters. On the other hand, H_2_S (either in the absence of any pro-inflammatory stimuli or when applied prior to LPS stimulation) enriches histone H3 methylation at the TNF-α and IL-6 promoters.

The importance of chromatin remodeling in the modulation of gene transcription has been investigated in a number of previous studies (25–29). The locus-specific changes in histone H3 observed in the present study and the associated suppression of inflammatory gene transcription may be one of the mechanisms responsible for the inhibition of cytokine production by H_2_S. Histone acetylation is often related to the chromatin openness, as it weakens the charge attraction between histones and DNA, leading to the decondensation of chromatin, thereby facilitating gene transcription. Both cytokine promoters analyzed in this study exhibited lower acetylation levels at histone H3 following treatment with NaHS. On the other hand, H3K27 trimethylation brings about transcriptional repression (31), as this epigenetic regulation is related to the silencing of human polycomb target genes (32). Treatment with the H_2_S donor alone or together with LPS increased H3K27 methylation at both the IL-6 and TNF-α promoters. A similar response was observed in the methylation of this histone at Lys9, which was enriched at the promoters analyzed in the groups that received NaHS or HaHS together with LPS. H3K9 methylation is linked to heterochromatin and to an endurance of transcriptional repression (33). Furthermore, it has been shown that H3K9 methylation acts as a regulatory mechanism for inducible inflammatory genes (33).

It has been demonstrated that, in unstimulated cells, H3K9 methylation is a mechanism for silencing the transcription of some genes whose expression rapidly increases following exposure to stimuli (33). On the other hand, in stimulated cells, H3K9 methylation may also repress inflammatory gene transcription (33). We found that H3K9me was associated with IL-6 but not with TNF-α in stimulated cells. This difference indicates a mechanism which allows for the rapid increase in IL-6 expression, as this cytokine can either function as a pro- or anti-inflammatory cytokin and it is important to the regulation of other inflammatory mediators following LPS stimulation.

In conclusion, the present study established a connection between H_2_S and epigenetic modulation. Future research on the mechanisms through which this action is associated with the various, previously demonstrated effects (12–16) of H_2_S on gene transcription, and inflammatory and cell growth signaling is required. In additition, whether endogenous H_2_S, which is similar to exogenous H_2_S used in the present study, modulates histones remains to be elucidated. Finally, the results of the present study remain to be confirmed under *in vivo* conditions. While much work remains to be done in this area of research, on the whole, our findings may prove to be beneficial for future studies exploring the the effects of H_2_S on epigenetic regulation.

## Abbreviations

BCA: bicinchoninic acid
ChIP: chromatin immuno-precipitation
H2S: hydrogen sulfide
H3K9: lysine 9 of histone H3
HAT: histone acetyltransferase
H3K27: lysine 27 of histone H3
HDAC: histone deacetylase
HMT: histone methyltransferase
IL-6: interleukin-6
LPS: lipopolysaccharide
NaHS: sodium hydrosulfide
PDF: polyvinylidene fluoride
PMA: phorbol myristate acetate
RIPA buffer: radioimmunoprecipitation assay buffer
SDS: sodium dodecyl sulfate
SEM: standard error of the mean
THP-1: Tamm-Horsfall protein 1
TNF-α: tumor necrosis factor-α
